# Brain–Heart Axis and Biomarkers of Cardiac Damage and Dysfunction after Stroke: A Systematic Review and Meta-Analysis

**DOI:** 10.3390/ijms21072347

**Published:** 2020-03-28

**Authors:** Chengyang Xu, Ang Zheng, Tianyi He, Zhipeng Cao

**Affiliations:** Department of Forensic Pathology, School of Forensic Medicine, China Medical University, Shenyang 110122, China; chengyangxv0404@hotmail.com (C.X.); ang.zheng@hotmail.com (A.Z.); tianyihe98@hotmail.com (T.H.)

**Keywords:** brain–heart axis, cardiac troponin, BNP, NT-proBNP, stroke, brain hemorrhage, ischemic stroke, meta-analysis

## Abstract

Background: Cardiac complications after a stroke are the second leading cause of death worldwide, affecting the treatment and outcomes of stroke patients. Cardiac biomarkers such as cardiac troponin (cTn), brain natriuretic peptide (BNP), and N-terminal pro-brain natriuretic peptide (NT-proBNP) have been frequently reported in patients undergoing a stroke. The aim of the present study is to meta-analyze the relationship between changes in such cardiac biomarkers and stroke and to present a systematic review of the previous literature, so as to explore the brain–heart axis. Methods: We searched four online databases pertinent to the literature, including PubMed, Embase, the Cochrane Library, and the Web of Science. Then, we performed a meta-analysis to investigate changes in cTn, BNP, and NT-proBNP associated with different types of stroke. Results and Conclusions: A significant increase in cTnI concentration was found in patients exhibiting a brain hemorrhage. BNP increased in cases of brain infarction, while the NT-proBNP concentration was significantly elevated in patients suffering an acute ischemic stroke and brain hemorrhage, indicating cardiac damage and dysfunction after a stroke. Our analysis suggests that several potential mechanisms may be involved in the brain–heart axis. Finally, clinicians should pay careful attention to monitoring cardiac function in the treatment of cerebrovascular diseases in order to provide a timely and more accurate treatment.

## 1. Introduction

Stroke, the second most common cause of death globally, is a medical emergency that represents the leading reason for adult disability [1,2]. With regard to the number of stroke survivors, this will continue to rise to 77 million by 2030, likely inducing more social and economic burdens [3]. Complications after a stroke, especially cardiac complications, frequently affect treatment and outcomes for patients [4,5,6]. Different types of cardiac complications have been frequently reported in stroke patients. The changes in the cardiovascular system affected by neurological disorders are defined as the brain–heart axis, which may lead to significant changes in heart function, reflected in the biochemical detection of cardiac biomarkers [7,8]. Therefore, biomarkers for cardiac injury and dysfunction are extremely crucial in indicating cardiovascular abnormalities after a stroke in order to guide better treatment.

Cardiac troponin (cTn), the brain natriuretic peptide (BNP), and the N-terminal pro-brain natriuretic peptide (NT-proBNP) are well-known biomarkers of cardiovascular disease [9,10,11,12,13]. As a regulatory protein for striated muscle contraction, cTn exists as a complex of myofibrils with three subunits, namely cardiac troponin T (cTnT), cardiac troponin I (cTnI), and cardiac troponin C (cTnC). Of these, cTnI and cTnT are cardiac-specific and highly sensitive biomarkers that reflect myocardial damage due to different etiologies [14]. Moreover, cTn has been recommended by the American Stroke Association as a routine test for patients suffering an acute stroke [15]. cTn is predominantly synthesized and secreted by ventricular cardiomyocytes under ventricular volume overload and elevated ventricular wall tension, and BNP and NT-proBNP are sensitive and of value in clinical practice for the diagnosis of cardiac dysfunction [11,16].

The manifestations of strokes can be divided into hemorrhagic and ischemic strokes [2]. Most strokes in the United States, approximately 87%, are ischemic. Intracerebral hemorrhages and subarachnoid hemorrhages (SAHs) account for approximately 10% and 3% of all strokes, respectively [17,18]. Clinical studies have shown that some patients with brain hemorrhages and cerebral ischemia develop cardiac abnormalities, with an increase in cTn, BNP, and NT-proBNP [15]. However, no study has meta-analyzed the relationship between the two kinds of stroke and various cardiac biomarkers (here, cTnI, cTnT, BNP, and NT-proBNP). Therefore, the present study is intended to meta-analyze the changes of biomarkers in response to cardiac injury and dysfunction, as well as to review the potential mechanisms of brain–heart interaction after a stroke in order to explore their values in the outcomes of strokes and to provide a reference for clinical practice.

## 2. Results

### 2.1. Study Selection

**Figure 1:**
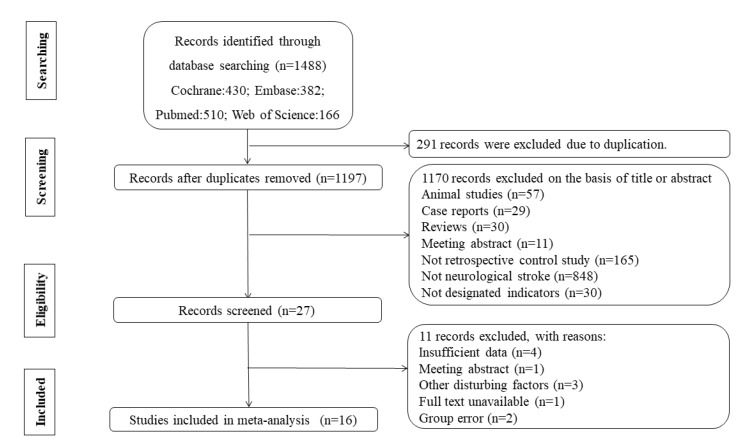
Flow diagram of the literature search process.

The search strategy yielded 1488 references, with 430 in Cochrane, 382 in Embase, 510 in PubMed, and 166 in the Web of Science (Figure 1). Of the 1488 articles, 291 records were excluded due to being duplicates. After title screening and detailed abstract evaluation, 27 studies were selected. In the end, 16 studies were selected for inclusion in this review, with a total of 1287 patients and 947 healthy people.

### 2.2. Data Extraction and Quality Assessment

Two independent reviewers extracted data using a simple, standardized template, shown in Table 1. The two reviewers assessed the quality of each included article using the Newcastle–Ottawa Quality Assessment Scale (NOS). All 16 records were considered as high-quality studies with a NOS score of ≥7 [14,19] (Table 2). The assessment criteria included the following: (1) selection: (a) adequate definition of cases, (b) representativeness of cases, (c) selection of controls, (d) and the interpretation of controls; (2) comparability: (e) comparability of cases and controls based on the design or analysis; (3) exposure: (f) ascertainment of exposure, (g) the same method of ascertainment for cases and controls, and (h) the non-response rate.

Two of the 16 included studies (Iltumur et al., 2006 [10], Altun et al., 2016 [20]) focused on the discrepancies of NT-proBNP and cTnI concentrations between patients in acute ischemic stroke and control groups with blood samples that were collected within 24 h. Two studies (Wang, 2009 [21], Baydin et al., 2015 [4]) compared cTnI concentrations between patients in brain hemorrhage and control groups. Of the 16 studies, only one study (Suleiman et al., 2017 [13]) measured and compared the cTnT concentration between ischemic stroke and control groups. Three studies (Kim et al., 2010 [22], Kim et al., 2012 [11], Ulvi et al., 2013 [23]) demonstrated differences in BNP levels in patients with acute cerebral infarction compared with those in the control group, while only one study (Li et al., 2010 [24]) compared BNP concentrations between brain hemorrhage and control groups. Seven of the 16 included studies (Makikallio et al., 2005 [25], Yuan et al., 2005 [26], Yip et al., 2006 [27], Fang et al., 2008 [12], Chen et al., 2012 [28], Altun et al., 2016 [20], Wang et al., 2018 [9]) compared NT-proBNP levels between those with a brain infarction and healthy people, while three (Fang et al., 2008 [12], Chen et al., 2012 [28], Niu et al., 2017 [16]) explicitly stated the discrepancies between brain hemorrhage and control groups.

### 2.3. Pooled Results from Literature-Based Meta-Analysis

**Figure 2:**
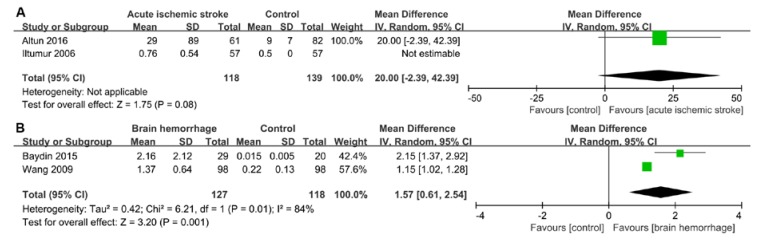
Forest plot of serum cTnI: (**A**) Comparison of serum cTnI between the acute ischemic stroke and control groups. (**B**) Comparison of serum cTnI between the brain hemorrhage and control groups. cTnI, cardiac troponin I; CI, confidence interval; IV, inverse variance; SD, standard deviation.

**Figure 3:**
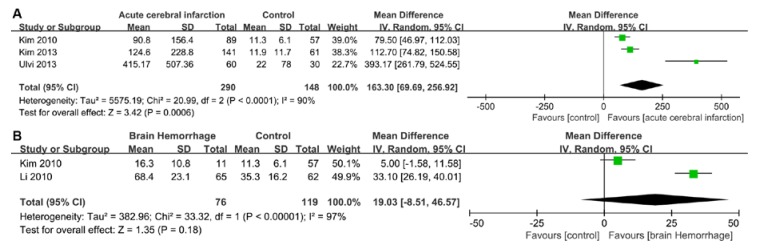
Forest plot of serum BNP: (**A**) Comparison of serum BNP between the acute ischemic stroke and control groups. (**B**) Comparison of serum BNP between the brain hemorrhage and control groups. BNP, brain natriuretic peptide; CI, confidence interval; IV, inverse variance; SD, standard deviation.

**Figure 4:**
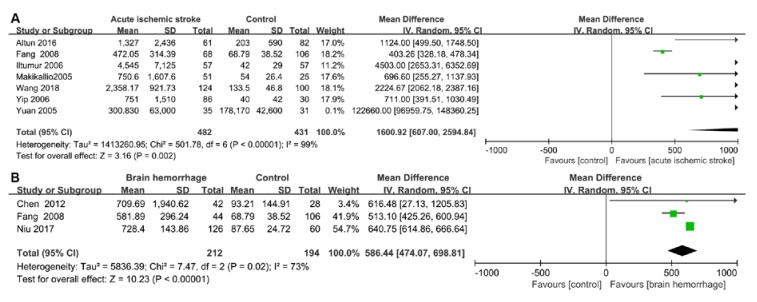
Forest plot of serum NT-proBNP: (**A**) Comparison of serum BNP between the acute ischemic stroke and control groups. (**B**) Comparison of serum BNP between the brain hemorrhage and control groups. BNP, brain natriuretic peptide; CI, confidence interval; IV, inverse variance; SD, standard deviation.

A total of 1287 stroke patients were recruited in the included articles. Heterogeneity was significant when *p* < 0.05 and/or I^2^ > 50%. Therefore, the random-effects model was used to analyze data in the six groups. It was found that cTnI was significantly raised in patients with a brain hemorrhage (weighted mean difference (WMD) = 1.57, 95% confidence interval (CI) = 0.61–2.54, *p* = 0.001, I^2^ = 84%, *p*-value of heterogeneity = 0.01), while there was no significant difference in cTnI between patients with ischemic stroke and a control group in one study (Iltumur et al., 2006 [10]) (WMD = 20.00, 95% CI = −2.39–42.39, *p* = 0.08) (Figure 2). However, due to the limited data in the literature, we did not conduct data analyses on cTnT. BNP concentrations were significantly increased in patients with acute cerebral infarction (WMD = 163.30, 95% CI = 69.69–256.92, *p* = 0.0006, I^2^ = 90%, *p*-value of heterogeneity <0.0001); however, no significant difference was found in the brain hemorrhage group (WMD = 19.03, 95% CI = −8.51–46.57, *p* = 0.18, I^2^ = 97%, *p*-value of heterogeneity <0.00001) (Figure 3). We believe that this result was found due to the small number of articles studied. Compared with the control group, the level of NT-proBNP in the acute ischemic stroke group was higher (WMD = 1600.92, 95% CI = 607.00–2594.84, *p* = 0.002, I^2^ = 99%, *p*-value of heterogeneity <0.00001). Additionally, a significant difference in NT-proBNP was found between patients in the brain hemorrhage and control groups (WMD = 586.44, 95% CI = 474.07–698.81, *p* <0.00001, I^2^ = 73%, *p*-value of heterogeneity = 0.02) (Figure 4).

## 3. Discussion

The main cause of death after a stroke is neurological damage, with cardiovascular complications being the second leading cause of death [29]. Increasingly, clinical and experimental evidence has shown a causal relationship between brain injury and cardiac dysfunction [13,30,31]. The present meta-analysis focused on differences in the levels of four cardiac biomarkers between stroke patients and healthy people. A significant increase in cTnI concentration was found in patients exhibiting a brain hemorrhage, while BNP increased in cases of brain infarction. The NT-proBNP concentration was found to be significantly elevated in patients with an acute ischemic stroke and brain hemorrhage. Our meta-analysis demonstrated that myocardial damage and cardiac dysfunction occurred after a stroke. Therefore, clinicians should take heed of cardiac biomarker levels and highlight the cardiac complications after a neurogenic stroke in order to develop better treatment strategies.

In the middle of the 19th Century, Barrow emphasized the connection between the brain and heart [32]. However, it was only in the last few decades that we have begun to understand the basic pathophysiology of the brain–heart axis. From our point of view, the brain–heart axis, focusing on the effects of changes in the nervous system on cardiac function, differs from the heart–brain axis, which predominantly emphasizes the neurological disorders induced by cardiac changes and injuries. Herein, we will discuss the cardiac damage induced by ischemic strokes and brain hemorrhages, respectively, as well as the potential mechanism of brain–heart interaction after a stroke.

### 3.1. Cardiac Damage Induced by Ischemic Stroke

The risk of cardiac complications in patients with an ischemic stroke is proportional to the severity of the stroke and neurological dysfunction. Similarly, impaired cardiac function following a severe acute ischemic stroke predicts worse functional outcomes and secondary complications [15]. It has been reported that 19.0% of patients had at least one serious adverse cardiac event within three months after an acute ischemic stroke, while 28.5% of patients showed left ventricular dysfunction with a left ventricular ejection fraction (LVEF) lower than 50% [33,34,35]. Worst of all, in the first three months after an acute ischemic stroke, 2%–6% of patients were reported to have died due to cardiac-related causes [33]. The electrocardiogram (ECG) results suggested that myocardial ischemia is very common in the acute phase of an ischemic stroke, with 30%–40% of ECG results showing ischemic changes at the baseline when patients with a known heart disease or use of cardiac medication were excluded [10,36,37].

As a sensitive biomarker, cTn clinically reflects myocardial damage. In the present study, in one of the two included studies that focused on cTnI levels after an acute ischemic stroke, the difference in cTnI between patients undergoing an acute ischemic stroke and healthy people could not be estimated according to the Review Manager software [10]. Though the difference in cTnI in acute ischemic stroke was not statistically significant (*p* = 0.08), one of the included studies highlighted how the level of cTnI in patients with an acute ischemic stroke was significantly higher than that in the control group (29 ± 89 vs. 9 ± 7 ng/mL). In addition, elevated cTn was observed in 5%–8% of patients with an acute ischemic stroke [13,20,38,39]. The LVEF (especially <40%) and cTn levels (especially >20 ng/mL) were associated with the severity of ischemic stroke, and these, plus the location of the lesion, were independent risk factors for mortality after suffering an acute ischemic stroke [40,41]. In addition, a prospective study pointed out that cTn positivity on admission was an independent prognostic predictor of an acute ischemic stroke [42].

According to the present meta-analysis, the NT-proBNP levels of patients with an acute ischemic stroke were significantly elevated when compared with the control group. It has been reported that the level of NT-proBNP increased within 24 h after the onset of an acute ischemic stroke and remained elevated during the six day study period [43]. A multivariate analysis demonstrated that the U.S. National Institutes of Health Stroke Scale (NIHSS) was one of the strongest independent predictors of increased NT-proBNP levels [27]. Furthermore, the increase in NT-proBNP (≥150 pg/mL) was the strongest independent predictive indicator of long-term unfavorable clinical outcomes in patients with an acute ischemic stroke [27]. An NT-proBNP level of 1583.50 pg/mL and an NIHSS score of >12.5 were independent factors associated with hospital death [27]. Therefore, myocardial damage and/or cardiac dysfunction may be induced by an acute ischemic stroke, pointing to the need for clinicians to be aware of cTn, as well as NT-proBNP levels, during admission and therapy.

### 3.2. Cardiac Damage induced by a Brain Hemorrhage

Brain hemorrhages mainly include intracerebral hemorrhages and SAHs. Intracerebral hemorrhages are the most common stroke subtype, accounting for 10%–15% of all strokes, with an estimated annual global incidence of 16 per 100,000 people [44,45,46]. About 4% of patients with an intracerebral hemorrhage experience a series of cardiac complications, such as acute myocardial infarction, ventricular fibrillation, acute heart failure, and cardiac death within two days, of which acute heart failure is the most common serious in-hospital cardiac event [47,48]. SAHs are mainly caused by ruptured aneurysms, with a worldwide incidence of approximately nine per 100,000 people and a mortality rate of about 60% at six months [49,50]. In addition to secondary nerve injury, SAHs are always associated with non-neurologic complications, such as neurocardiogenic injury, neurogenic pulmonary edema, hyperglycemia, and electrolyte imbalance, of which cardiac and pulmonary complications are the most common complications [51]. Neurocardiogenic injuries after an SAH include ECG abnormalities, arrhythmia, myocardial infarction, left ventricular dysfunction, and even cardiac arrest [4,52,53]. In a prospective study that included 580 SAH patients, ECG abnormalities were found in 40%–100% of patients, with the incidence of arrhythmia in patients with cerebral hemorrhages being 4.3% [54]. It was reported that 80% of patients developed ischemic ECG changes within a year after their intracerebral hemorrhage or SAH, which was associated with a poorer outcome [55]. Despite suffering a non-cardiac stroke, the left ventricular end-diastolic diameter of more than half of SAH patients increased [15]. In addition, different lesion locations lead to different degrees of cardiac complications. For instance, brain hemorrhages involving the insular lobe are often associated with bradycardia, heart blockages, and atrial or ventricular tachycardia [7,56].

Among all known biomarkers, cTn is a more sensitive and specific biomarker of myocardial injury after a brain hemorrhage [51,57]. In the present study, the concentration of cTnI in patients of the brain hemorrhage group was significantly higher than that in the control group, which indicates myocardial injury after stroke. Previous studies have shown that 17%–28% of patients with an SAH have elevated serum cTnI levels. The pathophysiological mechanism of increased cTn after an SAH differs from that of ischemic heart disease [58,59,60]. It has been found that cTn levels rise sharply within 24 h after the onset of an acute cerebral hemorrhage, remaining at a high level within 72 h, and then gradually decreasing [21]. Moreover, cTn leakage is associated with the severity and outcome of SAHs in patients according to the Hunt–Hess scale, which is used to assess the state of neural function [61,62]. A retrospective study of 617 consecutive patients with SAH also showed increased mortality in patients with high cTnI levels [53]. Therefore, serum cTn levels can be used as indicators to predict hospital mortality and poor prognosis and are available for risk stratification during intracerebral hemorrhage hospitalization [47].

According to the present study, BNP and NT-proBNP concentrations were also significantly increased after a brain hemorrhage, the secretion of which mainly occurred due to the stretching of the atrium rather than a direct effect due to a hypothalamic hemorrhage [63]. Meaudre et al. found that BNP levels increased in 25 out 31 (81%) patients undergoing an SAH, where they peaked on the second day (mean of 126 ng/mL) and gradually decreased on Day 7 [64,65]. The correlation between BNP and cTnI concentrations may be due to the release of norepinephrine in the myocardium, which may cause myocardial necrosis [66]. Elevated BNP levels were associated with early post-SAH myocardial necrosis, pulmonary edemas, and left ventricular systolic and diastolic dysfunction [63]. The higher the plasma NT-proBNP level at the onset of a stroke, the greater the nerve function defect score, with a greater chance of a worse outcome at the six month follow-up [28].

### 3.3. Mechanism of Brain–Heart Interaction after a Stroke

**Figure 5:**
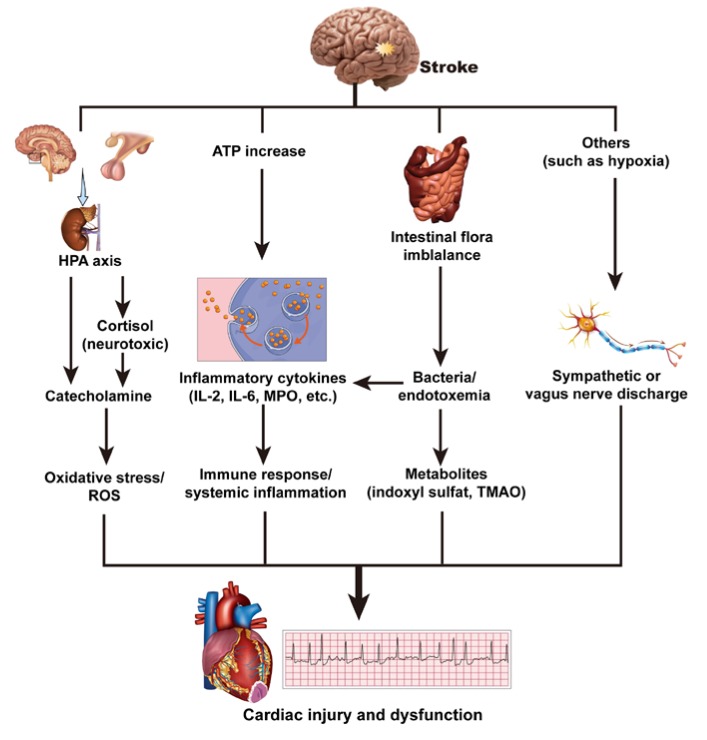
Summary of mechanisms for brain heart interaction after stroke. HPA axis, hypothalamic–pituitary–adrenal axis; MPO, myeloperoxidase; ROS, reactive oxygen species; TMAO, trimethylamine-N-oxide.

The potential mechanisms of the brain–heart axis resulting in cardiac injury and dysfunction after a stroke are discussed below and summarized in Figure 5.

#### 3.3.1. Hypothalamic–Pituitary–Adrenal Axis and Catecholamine Surge

According to data from animal models and clinical studies, increased catecholamine release is the most likely underlying cause of cardiac injury after a stroke, which may result in impaired systolic and diastolic function, abnormal repolarization, and myocardial injury [67,68]. The hypothalamic–pituitary–adrenal (HPA) axis, composed of three endocrine glands, namely the hypothalamus, pituitary, and adrenal glands, is the main regulator of body hormones that integrate mood, stress, physical activity, and metabolism [69]. Studies have demonstrated that serum levels of cortisol, which has been widely studied in relation to metabolism, inflammation, and cardiovascular disease, are associated with lesion location and stroke severity [70,71]. Sustained increases in cortisol, which are related to increased mortality after a stroke, may be neurotoxic [72]. Nerve injury causes the release of catecholamine in the circulation of myocardial nerve endings, which leads to cardiac toxicity, resulting in myocardial systolic dysfunction, myocardial cell necrosis, and apoptosis [10,15]. ECG changes are more visible in the anterior and lateral leads, usually in the presence of increased catecholamine release, and some can be prevented with antisympathetic drugs [7]. Veltkamp et al. proposed a model of cardiac change caused by cerebral ischemia, which can cause disturbances of catecholamine homeostasis in the heart. Cerebral ischemia leads to the upregulation of E3 ligase atrogin-1 and the upregulation of peroxisome proliferator-activated receptor gamma (PPARG)-dependent genes, resulting in myocardial cell atrophy and temporary cardiac dysfunction [73]. In addition, with the increase in sympathetic nerve activity and the enhanced release of catecholamines, aldosterone, and angiotensin II, the release of intracellular Ca^2+^ increases, as well as oxidative stress and the production of reactive oxygen species [7]. In this oxidizing environment, the combination of calcium and calmodulin results in the continued activation of calcium/calmodulin-dependent protein kinase II, which further promotes the progression of potential heart failure [74,75,76]. Therefore, as cardiac biomarkers, the levels of cTn and BNP are elevated after a stroke.

However, HPA axis activation and the adrenal cortex response both take time after a stroke, while cTn becomes elevated when irreversible myocardial damage occurs, which may be the reason why cTn response is small and insignificant in the early stages after a stroke [42,77]. Thus, a dynamic and continuous cardiac biological analysis is clinically recommended because of the slow HPA response interval.

#### 3.3.2. Immune Response and Systemic Inflammation

Inflammatory responses have been found to be associated with strokes [15,78,79]. After a stroke, neuronal and glial depolarization and damage to the plasma membrane of brain cells results in an increase in extracellular ATP, which can activate resident microglial cells and stimulate the production of inflammatory cytokines [80]. Spontaneous cerebral hemorrhages and ischemic strokes can activate the systemic inflammatory response [81]. SAHs, as a noninfectious injury, induce the systemic inflammatory response syndrome by triggering the activation of the immune system [82]. About 50% of patients with an SAH were found to have systemic inflammatory response syndrome on admission, and 85% of patients developed systemic inflammatory response syndrome within four days of admission to the hospital [83].

As markers and mediators of systemic inflammation, increases in inflammatory cytokines, such as interleukin (IL)-2, IL-6, myeloperoxidase, and integrins, are abundant in polymorphonuclear neutrophils and are related to oxidative stress after a stroke [80,84,85,86]. A large amount of evidence has shown an association between inflammatory cytokines and cardiovascular disease, including coronary artery disease, congestive heart failure, and myocardial ischemia/reperfusion-related injury/arrhythmia [85]. With the activation of leukocytes, inflammatory cytokines are secreted. In addition, the activity of inflammatory cytokines increases significantly, and they accumulate on endothelial cells and in subendothelial spaces, destroying the collagen layer in the atherosclerotic plaque, weakening fibrous envelopes, and eventually, leading to an acute coronary event [30,84]. Therefore, inflammation may play an important role in brain damage and heart dysfunction after suffering a stroke.

#### 3.3.3. Intestinal Flora Imbalance

The brain–gut and gut–heart axes are defined, respectively, as the interaction between the intestinal flora and central nervous system, as well as between the intestinal flora and heart [15]. The gut–blood barrier is responsible for the absorption of nutrients and water and prevents toxins and pathogenic microorganisms from entering the bloodstream [87].

In patients with a transient ischemic attack, a significant intestinal flora imbalance is manifested by an increase in opportunistic bacteria and a decrease in beneficial bacteria [88]. Furthermore, in an experiment in mice with transient focal cerebral ischemia, a decrease in intestinal permeability was positively correlated with stroke severity, leading to bacterial translocation, as well as the triggering of immune responses [89]. Bacterial metabolites were considered to mediate the communication between the gut microbiota and immune system, thus supporting the theory of inflammatory mechanisms [90]. After a stroke, with the change in intestinal permeability, bacteria and endotoxins are translocated to the bloodstream, and the increase in pro-inflammatory cytokines and systemic inflammation can induce or aggravate myocardial damage and cardiac dysfunction [91,92].

Intestinal microorganisms greatly influence the nature of blood metabolites, such as indoxyl sulfate [93]. In vitro studies have shown that indoxyl sulfate has a direct effect on cardiomyocytes by inducing hypertrophy and cardiac fibroblasts, which cause collagen synthesis. Indoxyl sulfate may possibly play an important role in heart remodeling in the activation of the p38 mitogen-activated protein kinase (MAPK), p42/44 MAPK, and nuclear factor kappa B pathways [94]. Mouse experiments have shown that the administration of indoxyl sulfate can induce left ventricular hypertrophy [95]. In addition, trimethylamine-N-oxide, a metabolite of intestinal flora, has been linked to cardiac dysfunction, heart attacks, and heart failure and enhances in vivo thrombosis [96]. Therefore, an imbalance of gut flora also plays a role in cardiac damage after a stroke.

#### 3.3.4. Others

Other potential mechanisms, such as hypoxia, have also been reported to be involved in the brain–heart axis. Cardiac pathophysiology after a stroke is believed to be caused by left ventricular myocardial ischemia, which may be induced by a coronary artery spasm or thrombosis and/or the mismatch of oxygen supply and demand in the context of hypertension and tachycardia [97]. The discharge of sympathetic or vagus nerves caused by hypoxia may be related to the ECG abnormalities in stroke patients [98,99]. However, because of the small number of related studies, further research is needed on cardiac damage caused by hypoxia after a stroke.

### 3.4. Limitations

The present study has several limitations. First, the heterogeneity of the present meta-analysis, which might be caused by different biochemical detection methods or different labs, cannot be ignored. Therefore, a random-effects model was used for the present meta-analysis, following the strategies in the Cochrane Handbook. Second, due to the limited number of articles, we did not specifically classify brain hemorrhages according to their cause and location, although some researchers have suggested that insular damage is more likely to cause cardiac damage [8]. Third, we did not perform a funnel plot due to the small number of included studies. Thus, the present meta-analysis is a pilot study to investigate the relationship between cardiac biomarkers and strokes. Associated animal and clinical studies are also needed to clarify the relationship and mechanism of cardiac damage and dysfunction after a stroke.

## 4. Materials and Methods

### 4.1. Searching Strategies

Two independent investigators (Xu and He) searched the PubMed, Embase, Cochrane Library, and Web of Science databases for studies focused on the relationship between strokes and cardiac biomarkers that were published before August 10, 2019. The manifestations of strokes can be divided into hemorrhagic and ischemic strokes and SAHs, as well as intracerebral hemorrhages, which belong to hemorrhagic strokes [17,18]. The following medical subject headings (MeSH terms) were used in the search string: (“intracranial hemorrhages” or “cerebral hemorrhage” or “subarachnoid hemorrhage” or “brain infarction”) and (“troponin” or “troponin I” or “troponin T” or “natriuretic peptide, brain” or “pro-brain natriuretic peptide (1–76)”) and (“control” or “group” or “groups” or “retrospective study”). Language restrictions were not imposed in the searching strategies. This procedure was repeated and checked until no further relevant articles were found.

### 4.2. Literature Screening and Selection Criteria

After removing duplicate references and filtering titles and abstracts, the two authors screened the full text of potentially relevant articles and resolved any divergence of opinion through discussion with a third investigator (He). Observational studies, which focused on the association between cTnI/cTnT/BNP/NT-proBNP and strokes, were included in the present study. Patients with a stroke, including intracerebral, subarachnoid hemorrhages, and cerebral ischemia, were all diagnosed by neuroimaging, such as computed tomographic scanning [100].

The following inclusion criteria were used to select the studies in the present study: (1) a retrospective and control study; (2) samples divided into stroke and healthy groups; (3) study focused on the concentration of cTn, BNP, or NT-proBNP; (4) adequate and reasonable data for the meta-analysis; and (5) samples in control groups with no history of neurological or cardiovascular disease and those in the normal range of an ECG and routine serum biochemistry.

The following criteria were used to exclude some of the literature: (1) a non-retrospective study or non-assigned group; (2) other disturbing factors; (3) insufficient data for the meta-analysis; (4) patients in experimental groups who included the following criteria: (1) younger than 18 years, (2) cardiac surgery, (3) cardiac pacing, (4) known arrhythmia, acute coronary syndromes, and myocardial infarction, (5) pheochromocytoma, or (6) renal impairment.

### 4.3. Data Extraction

A simple, standardized template was used by two independent reviewers to extract the data, including the author, year, experimental group data (disease, age, and amount), and control group data (age and amount). The quality of each included article was assessed by two independent investigators using the Newcastle–Ottawa Quality Assessment Scale (NOS). In cases of disagreement, a third investigator reviewed the article in question together with the other investigators until a consensus was reached.

### 4.4. Statistical Analysis

The Review Manager Version 5.3 software was used to perform the meta-analysis, and the strength of the association of biomarkers with stroke was measured by the WMD. Heterogeneity was assessed across all studies by Cochran’s Q-test and Higgins’ I^2^ value. The heterogeneity was significant if *p* < 0.05 and/or I^2^ > 50%. A random-effects model was used in the above situation, otherwise a fixed-effects model was used. Funnel plot and sensitivity analyses were not carried out due to the small number of included studies. *p* < 0.05 was considered statistically significant.

## 5. Conclusions and Perspectives

The present meta-analysis reviewed an increase in cardiac biomarkers after suffering an acute ischemic stroke and brain hemorrhage, indicating cardiac damage and dysfunction after the stroke. The biochemical analysis of cTn, BNP, and NT-proBNP can be used in formulating an auxiliary diagnosis, as indicators to reflect myocardial injury in stroke patients and to identify patients at high risk of a cardiac event. Clinicians should pay attention to monitoring cardiac biomarkers while focusing on the treatment of cerebrovascular diseases in order provide a more timely and accurate treatment.

## Figures and Tables

**Table 1:** Overview of included articles. Main data collected from each of the 16 included articles.

Author/Year	Marker	Experimental Groups			Control Groups	
		Disease	Age	n	Age	n
Makikallio et al., 2005 [25]	NT-proBNP	Brain infarction	67 ± 10	51	—	25
Yuan et al., 2005 [26]	NT-proBNP	Brain infarction	56 (50–67)	35	57 (53–69)	31
Iltumur et al., 2006 [10]	NT-proBNP, cTnI	Acute ischemic stroke	64.5 ± 11.3	57	61.3 ± 6.09	57
Yip et al., 2006 [27]	NT-proBNP	Acute ischemic stroke	66.0 ± 11.0	86	64.6 ± 6.5	30
Fang et al., 2008 [12]	NT-proBNP	Brain infarction	—	68	54.36 ± 8.29	106
Fang et al., 2008 [12]	NT-proBNP	Brain hemorrhage	—	44	54.36 ± 8.29	106
Wang, 2009 [21]	cTnI	Acute cerebral hemorrhage	62.5 (45–80)	98	63.6 (43–78)	98
Li et al., 2010 [24]	BNP	Cerebral hemorrhage	57 ± 10	65	56 ± 8	62
Kim et al., 2010 [22]	BNP	Acute ischemic stroke	66.6 ± 11.8	89	43.8 ± 12.0	57
Kim et al., 2010 [22]	BNP	Cerebral hemorrhage	56.4 ± 14.5	11	43.8 ± 12.0	57
Chen et al., 2012 [28]	NT-proBNP	Acute intracerebral hemorrhage	67 ± 13	42	57 ± 17	28
Kim et al., 2012 [11]	BNP	Acute cerebral infarction	67.6 ± 11.6	141	65.5 ± 7.3	61
Ulvi et al., 2013 [23]	BNP	Acute cerebral ischemia	68.5 ± 14.2	60	60.2 ± 12.4	30
Baydin, et al., 2015 [4]	cTnI	Nontraumatic SAH	58.8 ± 13.0	29	47.3 ± 8.6	20
Altun et al., 2016 [20]	NT-proBNP, cTnI	Acute ischemic stroke	71.4 ± 11	61	68.6 ± 8	82
Niu et al., 2017 [16]	NT-proBNP	Acute intracerebral hemorrhage	63.80 ± 8.46	126	63.52 ± 8.60	60
Suleiman et al., 2017 [13]	cTnT	Ischemic stroke	59 ± 14.08	100	59 ± 13.91	100
Wang et al., 2018 [9]	NT-proBNP	Acute cerebral infarction	67.4 ± 7.2	124	68.2 ± 8.1	100

NT-proBNP, N-terminal fragment of B-type natriuretic peptide; cTnI, cardiac troponin I; BNP, B-type natriuretic peptide; cTnT, cardiac troponin T; —, information not available; SAH, subarachnoid hemorrhage.

**Table 2:** Quality assessment of the included studies.

Study	Quality Indicators from the Newcastle–Ottawa Scale								
	Selection				Outcome Assessment ^1^	Exposure			Scores
	(a)	(b)	(c)	(d)	(e)	(f)	(g)	(h)	
Makikallio et al., 2005 [25]	☆	☆	☆	☆	☆	☆	☆		7
Yuan et al., 2005 [26]	☆	☆	☆	☆	☆	☆	☆		7
Iltumur et al., 2006 [10]	☆	☆	☆	☆	☆☆	☆	☆		8
Yip et al., 2006 [27]	☆	☆	☆	☆	☆☆	☆	☆		8
Fang et al., 2008 [12]	☆	☆	☆	☆	☆☆	☆	☆		8
Wang, 2009 [21]	☆	☆	☆	☆	☆☆	☆	☆		8
Li et al., 2010 [24]	☆	☆	☆	☆	☆	☆	☆		7
Kim et al., 2010 [22]	☆	☆	☆	☆	☆☆	☆	☆		8
Chen et al., 2012 [28]	☆	☆	☆	☆	☆☆	☆	☆		8
Kim et al., 2012 [11]	☆	☆	☆	☆	☆	☆	☆		7
Ulvi et al., 2013 [23]	☆	☆	☆	☆	☆☆	☆	☆		8
Baydin et al., 2015 [4]	☆	☆	☆	☆	☆☆	☆	☆		8
Altun et al., 2016 [20]	☆	☆	☆	☆	☆☆	☆	☆		8
Niu et al., 2017 [16]	☆	☆	☆	☆	☆☆	☆	☆		8
Suleiman et al., 2017 [13]	☆	☆	☆	☆	☆	☆	☆		7
Wang et al., 2018 [9]	☆	☆	☆	☆	☆	☆	☆		7

^1^ A maximum of two stars can be allotted in this category, one for study controls for the experimental and control groups, the other for a blood collection time of <24 h.

## Abbreviations

cTn	Cardiac troponin
BNP	Brain natriuretic peptide
NT-proBNP	N-terminal pro-brain natriuretic peptide
cTnT	Cardiac troponin T
cTnI	Cardiac troponin I
cTnC	Cardiac troponin C
NOS	Newcastle–Ottawa Quality Assessment Scale
SAH	Subarachnoid hemorrhage
WMD	Weighted mean difference
CI	Confidence interval
ECG	Electrocardiogram
LVEF	Left ventricular ejection fraction
NIHSS	U.S. National Institutes of Health Stroke Scale
HPA	Hypothalamic–pituitary–adrenal
PPARG	Peroxisome proliferator-activated receptor gamma
MPO	Myeloperoxidase
ROS	Reactive oxygen species
TMAO	Trimethylamine-N-oxide
IL	Interleukin
MAPK	Mitogen-activated protein kinase
MeSH	Medical subject headings
